# ER-α36, a Novel Variant of ER-α, Mediates Estrogen-Stimulated Proliferation of Endometrial Carcinoma Cells via the PKCδ/ERK Pathway

**DOI:** 10.1371/journal.pone.0015408

**Published:** 2010-11-04

**Authors:** Jing-Shan Tong, Qing-Hua Zhang, Zhen-Bo Wang, Sen Li, Cai-Rong Yang, Xue-Qi Fu, Yi Hou, Zhao-Yi Wang, Jun Sheng, Qing-Yuan Sun

**Affiliations:** 1 College of Life Sciences, Jilin University, Changchun, China; 2 State Key Laboratory of Reproductive Biology, Institute of Zoology, Chinese Academy of Sciences, Beijing, China; 3 Department of Medical Microbiology and Immunology, Creighton University Medical School, Omaha, Nebraska, United States of America; 4 Yunnan Agricultural University, Kunming, China; Health Canada, Canada

## Abstract

**Background:**

Recently, a variant of ER-α, ER-α36 was identified and cloned. ER-α36 lacks intrinsic transcription activity and mainly mediates non-genomic estrogen signaling. The purpose of this study was to investigate the function and the underlying mechanisms of ER-α36 in growth regulation of endometrial Ishikawa cancer cells.

**Methods:**

The cellular localization of ER-α36 and ER-α66 were determined by immunofluorescence in the Ishikawa cells. Ishikawa endometrial cancer control cells transfected with an empty expression vector, Ishikawa cells with shRNA knockdown of ER-α36 (Ishikawa/RNAiER36) and Ishikawa cells with shRNA knockdown of ER-α66 (Ishikawa/RNAiER66) were treated with E2 and E2-conjugated to bovine serum albumin (E2-BSA, membrane impermeable) in the absence and presence of different kinase inhibitors HBDDE, bisindolylmaleimide, rottlerin, H89 and U0126. The phosphorylation levels of signaling molecules and cyclin D1/cdk4 expression were examined with Western blot analysis and cell growth was monitored with the MTT assay.

**Results:**

Immunofluorescence staining of Ishikawa cells demonstrated that ER-α36 was expressed mainly on the plasma membrane and in the cytoplasm, while ER-α66 was predominantly localized in the cell nucleus. Both E2 and E2-BSA rapidly activated PKCδ not PKCα in Ishikawa cells, which could be abrogated by ER-α36 shRNA expression. E2-and E2-BSA-induced ERK phosphorylation required ER-α36 and PKCδ. However, only E2 was able to induce Camp-dependent protein kinase A (PKA) phosphorylation. Furthermore, E2 enhances cyclin D1/cdk4 expression via ER-α36.

**Conclusion:**

E2 activates the PKCδ/ERK pathway and enhances cyclin D1/cdk4 expression via the membrane-initiated signaling pathways mediated by ER-α36, suggesting a possible involvement of ER-α36 in E2-dependent growth-promoting effects in endometrial cancer cells.

## Introduction

Endometrial cancer is one of the most common female pelvic malignancies and is the fourth most common type of cancer in North American and European women [1], [2]. It is well-known that the steroid hormone 17β-estradiol (E2) plays an important role in the development of endometrial carcinoma [3], [4]. In the classical model, E2 regulates the expression of estrogen responsive genes by binding to the estrogen receptor-α (ER) located in the cell cytoplasm, and ligand-bound receptors then migrate to the nucleus and regulate the transcription of target genes via binding to the estrogen responsive elements (EREs) within the target gene promoter [5], [6]. However, accumulating evidence indicated that ER-α also exists on the plasma membrane and participates in rapid estrogen signaling or membrane-initiated estrogen signaling. It has been reported that ER-α is modified by posttranslational palmitoylation in the ligand-binding domain that may contribute to its membrane localization [7]. Previously, we identified and cloned a variant of ER-α with a molecular weight of 36 kDa that is transcribed from previously unidentified promoter located in the first intron of the original 66 kDa ER-α (ER-α66) gene [8]. ER-α36 lacks both transcriptional activation domains of ER-α66 (AF-1 and AF-2), but it retains the DNA-binding domain and partial ligand-binding domain. It possesses a unique 27 amino acid domain that replaces the last 138 amino acids encoded by exons 7 and 8 of the ER-α66 gene.

PKC isoforms are involved in a variety of cellular functions, including growth, differentiation, tumor promotion, aging, and apoptosis [9], [10], [11]. The PKC family consists of several subfamilies; depending on differences in their structure and substrate requirements 1) classical (α,βI,βII and γ), all of which are activated by calcium and diacylglycerol (DAG); 2) novel (δ, ε, η and θ), all of which require DAG but are calcium-insensitive; 3) atypical (ζ and λ/ι), which are not responsive to either DAG or calcium [9], [12], [13]. It has been reported that E2 rapidly increases PKC activity via a membrane pathway not involving both ER-α or ER-β [14]. Our previous report demonstrated that 17β-estradiol induced the activation the MAPK/ERK pathway and stimulated the cells proliferation through the membrane-based ER-α36 [15]. We thus hypothesized that ER-α36 may be also involved in the E2-induced PKC activation.

In the present study, we studied the ER-α36 function in endometrial cancer cells and found that ER-α36 mediates E2 induced the membrane-associated PKCδ and the MAPK/ERK pathways leading to modulation of growth and survival of endometrial carcinoma cells.

## Results

### Differential expression of ER-α36 and ER-α66 in Ishikawa cells

**Figure 1:**
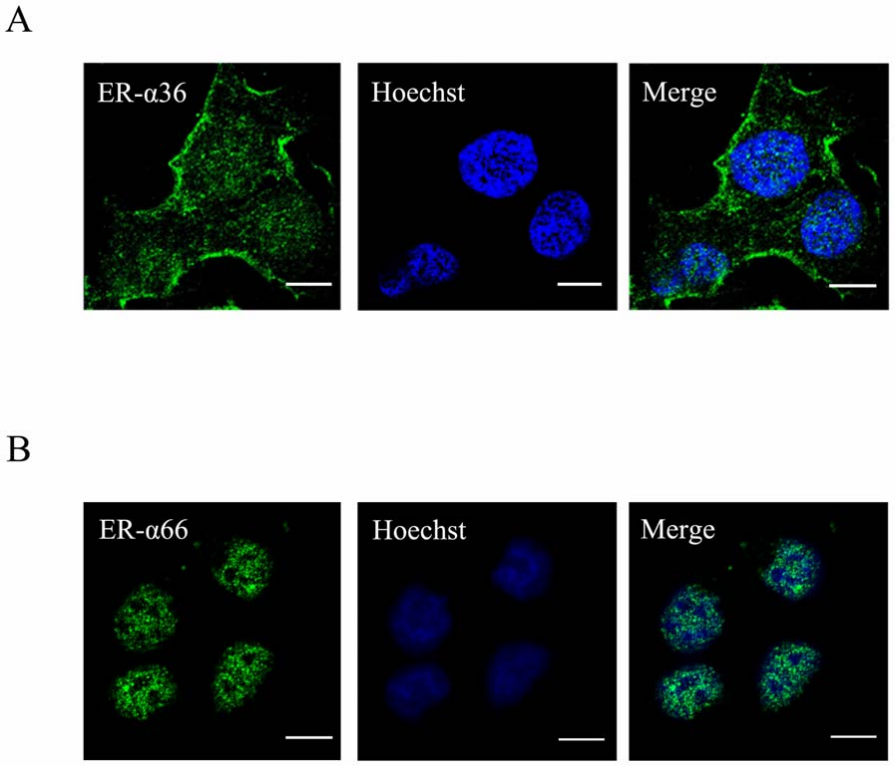
Subcellular localization of ER-α36 and ER-α66 in Ishikawa cells. A. Ishikawa cells cultured on coverslips were fixed and immunofluorescently stained with the anti-ER-α36 specific antibody (green). The cells were also stained with Hoechst 33258 (blue) to show the cell nuclei. B. ER-α66 expression detected by immunofluorescence in Ishikawa cells. The nucleus was stained by Hoechst 33258. Bar, 10 micrometers.

ER-α36 is a variant of ER-α generated by alternative promoter usage and alternative splicing [8]. To examine ER-α36 localization in Ishikawa cells, the indirect immunofluorescence assay was performed with anti-ER-α36 specific antibody raised against the 20 amino acids at the *C*-terminal of ER-α36 that are unique to ER-α36 [15]. Immunofluorescent staining revealed that ER-α36 was expressed on the plasma membrane and in the cytoplasm of Ishikawa cells (Fig. 1A) while ER-α66 was predominantly localized in the cell nucleus (Fig. 1B).

### E2 and E2-BSA rapidly induces the activation of PKCδ in Ishikawa cells

**Figure 2:**
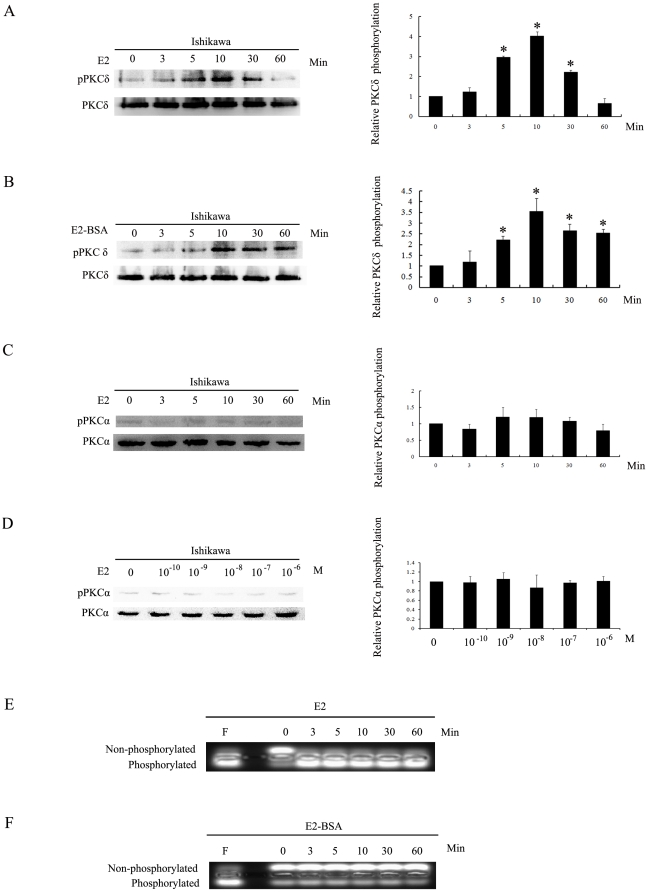
Effects of E2 and E2-BSA on the activation of PKCδ, PKCα and PKA in Ishikawa cells. A and B. Serum starved Ishikawa cell was treated with 10 nM E2 or 10 nM E2-BSA for the indicated time points. Protein extracts were prepared and used for Western blot analysis to measure levels of PKCδ phosphorylation. Protein levels of total PKCδ were also examined as controls. Each bar represents mean value ± SEM (n = 3). *, P<0.05 compared to untreated cells. C. Serum starved Ishikawa cell was treated with 10 nm E2 for the indicated time points and cells were lysed for western blot analysis to measure levels of PKCα phosphorylation. Protein levels of total PKCα were measured as controls. D, Serum starved Ishikawa cell was treated with 0, 0.1,1, 10, 100 and 1,000 nM E2 for 20 min. Protein extract was prepared for Western blot analysis to measure levels of PKCα phosphorylation and total PKCα. E and F, Serum starved Ishikawa cells were treated with 10 nM E2 or 10 nM E2-BSA for indicated time points, 20 µM Forskolin (F) was added for 15 min as a positive control, after which the cells were lysed and tested in the PepTag PKA assay. Samples were separated on an agarose gel. The lower band represents phosphorylated peptide, and the upper band represents the remaining unphosphorylated peptide.

We first examined PKC activation by E2 and E2-BSA in Ishikawa cells. Cells treated with E2 (Fig. 2A) showed a rapidly response in the phosphorylation of PKCδ within 5 min of exposure, while the total PKCδ amount was without any change, indicating that E2 induced PKCδ phosphorylation. The membrane impermeable E2-BSA (Fig. 2B) also elicited a similarly rapidly phosphorylation of PKCδ, which then declined gradually in approximately 60 min. These results demonstrated that both E2 and E2-BSA were able to rapidly activate PKCδ in Ishikawa cells. Similar time course studies were performed for E2-induced phosphorylation of PKCα in the Ishikawa cells (Fig. 2C). The concentration-response studies for the PKCα revealed that the E2 could not induce changes of PKCα phosphorylation even at 1 uM (Fig. 2D).

### E2, but not E2-BSA, induces PKA activation in Ishikawa cells

We then examine whether E2 and E2-BSA stimulate cAMP-dependent protein kinase (PKA) activation in Ishikawa cells. As shown in Fig. 2E, E2 treatment resulted in rapidly increase of phosphorylation level of PKA within 3 min, and treatment of the cells with Forkolin (20 µM) for 15 min (F) also induced PKA phosphorylation. However, when cells were treated with E2-BSA (Fig. 2F), no significant change in the phosphorylation of PKA was observed, indicating that E2 induces PKA signaling not from the plasma membrane.

### ER-α36 but not ER-α66 mediates E2-BSA-stimulated PKCδ activation

**Figure 3:**
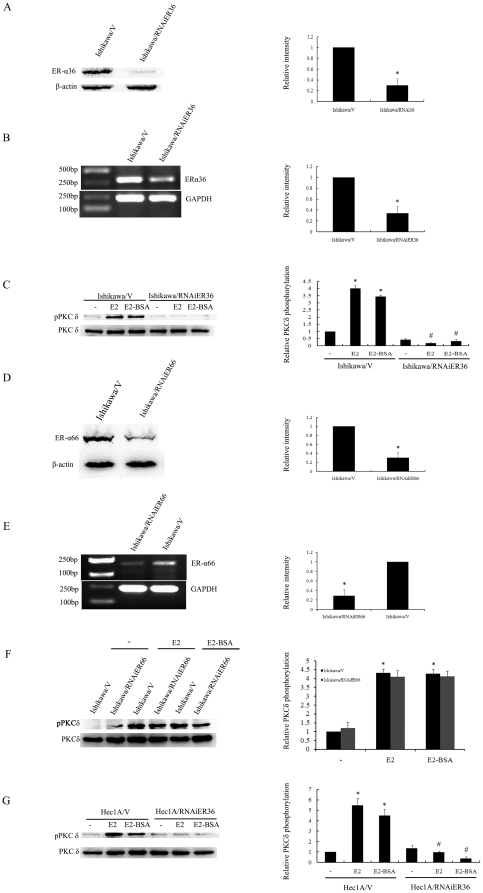
ER-α36 mediates E2-stimulated PKCδ activation. A and B. ER-α36 expression in Ishikawa/V and Ishikawa/RNAiER36 cells. Each bar represents mean value ± SEM (n = 3). *, P<0.05 compared to Ishikawa/V cells. C. Ishikawa/V and Ishikawa/RNAiER36 cells were treated with 10 nm E2 or 10 nm E2-BSA for 10 min, and PKCδ phosphorylation was analyzed by Western blot. Total levels of PKCδ were measured as controls, and each bar repents mean value ± SEM (n = 3). *, P<0.05 compared to untreated cells. #, P<0.05 compared to E2- or E2-BSA- treated Ishikawa/V cells. D and E. ER-α66 expression in Ishikawa/V and Ishikawa/RNAiER66 cells. Each bar represents mean value ± SEM (n = 3). *, P<0.05 compared to Ishikawa/V cells. F. Ishikawa/V and Ishikawa/RNAiER66 cells were treated with 10 nM E2 or 10 nM E2-BSA for 10 min, and then PKCδ phosphorylation was assessed with Western blot. Total PKCδ was measured as controls. Each bar represents mean value ± SEM (n = 3). *, P<0.05 compared to untreated cells. G, Hec1A/V and Hec1A/RNAiER36 cells were treated with 10 nM E2 or 10 nM E2-BSA for 10 min, and PKCδ phosphorylation was analyzed by Western blot. Total PKCδ was measured as controls. Each bar represents mean value ± SEM (n = 3).*, P<0.05 compared to untreated cells. #, P<0.05 compared to E2- or E2-BSA-treated Hec-1A/V cells.

To determine the involvement of ER-α36 in E2 activity observed in Ishikawa cells, we decided to knockdown ER-α36 expression with the shRNA approach. We established a stable cell line that expresses shRNA specifically against the unique 3′UTR of ER-α36 (Ishikawa/RNAiER36), and found that ER-α36 expression was dramatically down-regulated in the cells (Figs. 3A & 3B). As shown in Fig. 3C, E2 and E2-BSA failed to induce PKCδ phosphorylation in Ishikawa/RNAiER36 cells. Next, we knockdown ER-α66 expression with shRNA in Ishikawa cells (Figs. 3D & 3E). Both E2 and E2-BSA induced phosphorylation of PKCδ in the Ishikawa cells with ER-α66 expression knocked-down with shRNA (Fig. 3F).

To confirm the results, we used endometrial cancer Hec1A cells, which have been reported are an ER-α66 negative cell line [16], and Hec1A/RNAiER36, which have been described in our previous report [17]. The knockdown of ER-α36 expression was able to abrogate both E2 and E2-BSA induced (Fig. 3G) in Hec1A cells, indicating the involvement of ER-α36 in E2 induced-PKCδ phosphorylation. Thus, our data strongly demonstrate that ER-α36 not ER-α66 is involved in E2-induced PKCδ activation in endometrial cancer cells.

### E2 activates ERK1/2 through the PKCδ signaling pathway

**Figure 4:**
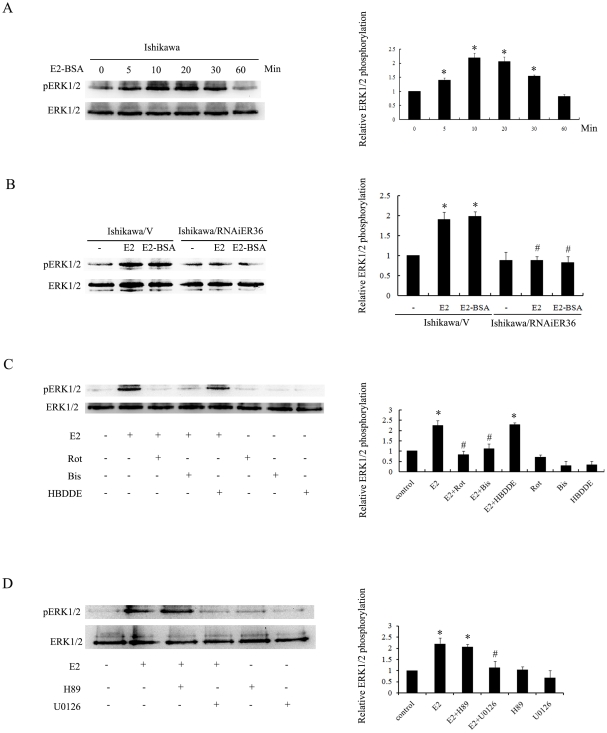
E2 activates the ERK1/2 through the PKCδ signaling pathway. A. Serum starved Ishikawa cells were treated with E2-BSA for indicated time points and cell lysates were immunoblotted with antibody against phosphorylated ERK1/2. Levels of phosphorylation were normalized with the total ERK1/2 protein, and each bar represents means value ± SEM (n = 3). *, P<0.05 compared to untreated cells. B. Ishikawa/V and Ishikawa/RNAiER36 cells were treated with 10 nM E2 or 10 nM E2-BSA for 10 min, and ERK1/2 phosphorylation was analyzed with Western blot. Expression was normalized to total ERK1/2, and each bar represents mean value ± SEM (n = 3).*, P<0.05 compared to untreated cells. #, P<0.05 compared to E2- or E2-BSA-treated Ishikawa/V cells. C. Serum starved Ishikawa cells were treated with 10 nM E2 or together with 5 µM of PKCδ specific inhibitor rottlerin, 5 µM of pan-PKC inhibitor bisindolylmaleimide or 40 µM of PKCα specific inhibitor HBDDE. ERK1/2 phosphorylation was analyzed with Western blot. Expression was normalized to total ERK1/2, and each bar represents mean value ± SEM (n = 3). *, P<0.05 compared to untreated cells. #, P<0.05 compared to E2-treated Ishikawa cells. D. Serum starved Ishikawa cells were treated with 10 nM E2 or together with 5 µM of PKA specific inhibitor H89 or 10 µM of MEK inhibitor U0126, and ERK1/2 phosphorylation was analyzed by Western blot. Expression was normalized to total ERK1/2, and each bar represents mean value ± SEM (n = 3). *, P<0.05 compared to untreated cells. #, P<0.05 compared to E2-treated Ishikawa cells.

Our previous data demonstrated that 17β-estradiol also induced the activation of the MAPK/ERK and stimulate the cells proliferation through the membrane-based ER-α36 [15]. Here we tested whether ER-α36 also mediates E2-BSA induced ERK1/2 activation in Ishikawa cells. As shown in Fig. 4A, E2-BSA treatment induced the rapid phosphorylation of ERK1/2 in Ishikawa cells while Ishikawa cells with ER-α36 expression knocked down with the shRNA failed to response to either E2 or E2-BSA indicating the involvement of ER-α36 in estrogen stimulated ERK1/2 activation (Fig. 4B). We then investigated whether E2-induced activation of ERK1/2 in Ishikawa cells requires activation of PKC or PKA. Ishikawa cells were treated with E2 in the presence or absence of the nonspecific PKC inhibitor bisindolylmaleimide (Bis), a PKCδ specific inhibitor rottlerin (Rot), a PKCα specific inhibitor HBDDE. Treatment of Bis and rottlerin strongly inhibited E2-induced ERK1/2 activation, indicating the involvement of PKCδ in E2-induced ERK1/2 activation (Fig. 4C). However, both HBDDE and H89 (Fig. 4D) had no effect on E2-induced ERK1/2 activation. Our results thus indicated that the ER-α36-mediated activation of PKCδ is required for activation of the ERK1/2 pathway.

### ER-α36 is involved in regulation of cyclin D1 protein expression in Ishikawa cells

**Figure 5:**
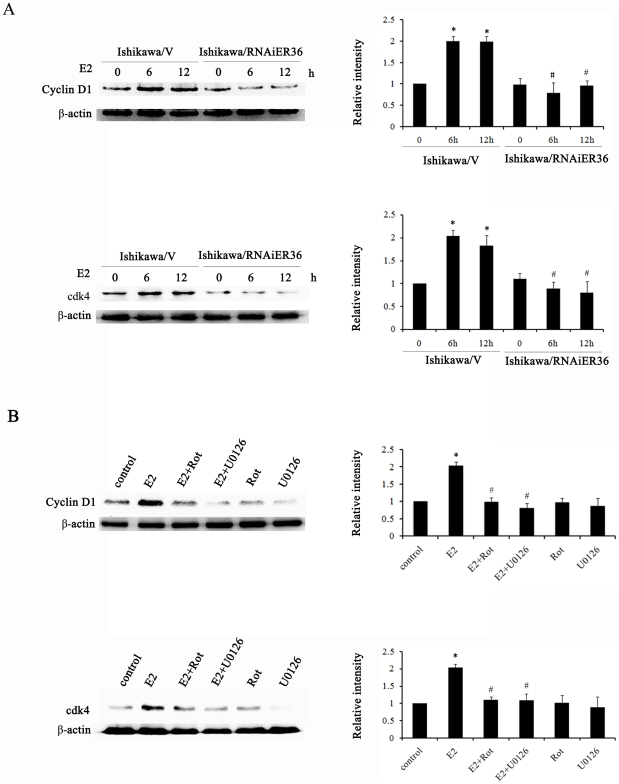
ER-α36 mediates E2-stimulated cyclin D1 expression. A. Western blot analysis of cyclin D1 and cdk4 expression in Ishikawa/V and Ishikawa/RNAiER36 cells treated with 10 nM E2 for 6 h and 12 h. Levels of cyclin D1 and cdk4 expression were normalized to the levels of β-actin, and each bar represents mean value ± SEM (n = 3). *, P<0.05 compared to untreated cells. #, P<0.05 compared to E2-treated Ishikawa/V cells. B. Ishikawa cells were treated for 12 h with E2 or together with 5 µM of PKCδ inhibitor rottlerin or 10 µM MEK inhibitor U0126. Levels of cyclin D1 and cdk4 expression were normalized to the levels of β-actin, and each bar represents mean value ± SEM (n = 3). *, P<0.05 compared to untreated cells. #, P<0.05 compared to E2-treated Ishikawa cells.

Cyclin D1 together with its binding partners cyclin dependent kinase 4 and 6 (cdk4 and cdk6) forms activation complexes that promote cell cycle progression and can function as a transcription coregulater. Overexpression of cyclin D1 is involved in endometrial carcinogenesis [18]. The E2-induced proliferation was associated with the up-regulation of cyclin D1 [19], [20], [21]. We measured the expression levels of cyclin D1 and cdk4 in Ishikawa cells treated with E2. As shown in Fig. 5A, treatment with E2 induced cyclin D1 and cdk4 expression in Ishikawa/V cells but not in Ishikawa/RNAiER36 cells, which could be effectively abrogated by the PKCδ inhibitor rottlerin and MEK inhibitor U0126 (Fig. 5B). Taken together, these results indicate that ER-α36 mediates E2-induced activation of the PKCδ/ERK pathway and cyclin D1 expression.

### Cell proliferation

**Figure 6:**
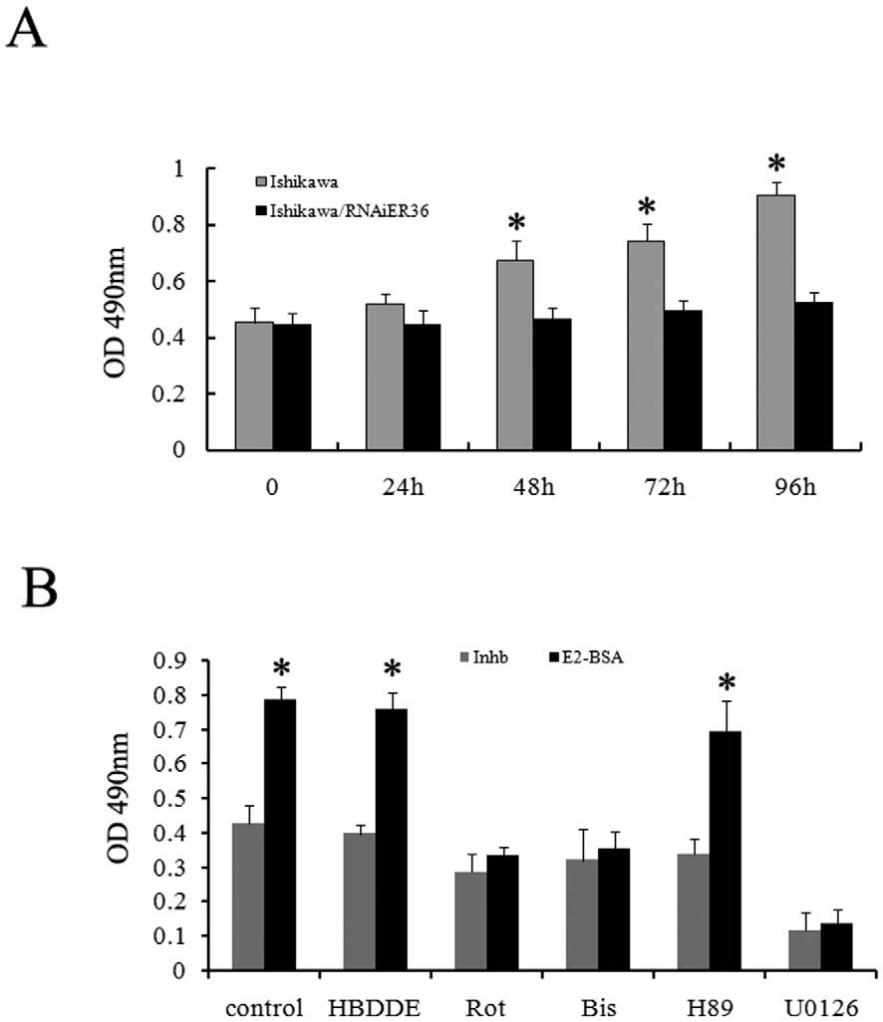
ER-a36 mediates E2 stimulated cell proliferation. A. Ishikawa/V and Ishikawa/RNAiER36 cells were treated with 10 nM E2-BSA for 24 h, 48 h, 72 h and 96 h. MTT assay was performed as described in the materials and methods. Results of three independent experiments were averaged and mean value ± SEM are shown. *, P<0.05 compared to E2-BSA treated Ishikawa/V cells respectively. B. Ishikawa cells were treated with 10 nM E2-BSA alone or together with 5 µM rottlerin, a PKCδ specific inhibitor, or 5 µM bisindolylmaleimide, a pan-PKC inhibitor, or HBDDE, a PKCα specific inhibitor, or 5 µM H89, a PKA specific inhibitor for 72 h, and MTT assays were then performed. Results of three independent experiments were averaged and mean value ± SEM are shown. *, P<0.05 compared to control cells.

To further investigate the role of ER-α36-mediated estrogen signaling in cell proliferation of endometrial cancer cells, Ishikawa/V and Ishikawa/RNAiER36 cells were treated with E2-BSA and cell growth was measured by the MTT assay. MTT assay shown that E2-BSA stimulated growth of Ishikawa/V cells while had no effect on growth of Ishikawa/RNAiER36 cells (Figs. 6A & B). Bisindolylmaleimide (a general PKC inhibitor), rottlerin (a PKCδ specific inhibitor) or U0126 (a MEK specific inhibitor) were able to inhibit E2-BSA-induced cell proliferation. However, HBDDE (a PKCα specific inhibitor) and H89 (a PKA specific inhibitor) failed to inhibit E2-BSA-induced cell proliferation. These results again suggest that E2-BSA-induced cell proliferation is predominantly mediated through the ER-α36/PKCδ/ERK pathway in endometrial cancer cells.

## Discussion

Estrogen receptors are members of the nuclear receptor superfamily and function as ligand-dependent transcription factors in the nucleus to mediate estrogen signaling. However, accumulating evidence indicated the existence of rapid signaling responses to E2 that are independent of transcriptional effects [22], [23]. Previously, a novel variant of ER-α, ER-α36, was identified, cloned and characterized [8]. ER-α36 is expressed in established ER-positive and ER-negative breast cancer cells and specimens from breast cancer patients that lacks expression of ER-α66 [24], suggesting that ER-α36 expression is regulated differently from ER-α66. In this study, confocal microscopy results show that ER-α36 is expressed on the plasma membrane and in the cytoplasm of Ishikawa cells. In contrast, ER-α66 is predominantly localized in the cell nucleus.

Previous studies have shown that ER-α36 mediates membrane-initiated effects of estrogen signaling by activation of the mitogen activated protein kinase (MAPK) signaling pathway, and stimulates cell growth [15]. ER-α36 also mediates activation of the MAPK/ERK signaling induced by antiestrogens, such as tamoxifen and ICI 182,780[15], [25]. In addition, ER-α36 mediates membrane-initiated testosterone signaling by activation of the ERK and AKT signaling pathway [17]. Recently, investigators have recognized rapid non-genomic effects of estrogen on several cellular processes, such as activation of PKC, PKA, calcium channel, and AKT to stimulate physiological effects [26], [27], [28], but the mechanisms are not well understood and have not been extensively studied [29], [30], [31]. PKC family consists of a number of serine-threonine kinases that are divided into three groups based on their activating factors. PKC activators act as tumor promoters. Although early studies of the effect of PKCδ on cell proliferation suggested that PKCδ suppresses proliferation [32], [33], [34]. However, several reports have demonstrated that PKCδ could act as a positive regulator of cell proliferation [35], [36], [37], [38]. Here, we found that both E2 and E2-BSA stimulated the activation of PKCδ signaling pathway in Ishikawa cells, and knockdown of ER-α36 expression with the shRNA abrogated E2-induced PKCδ phosphorylation. These indicated that ER-α36 mediates membrane-initiated PKCδ pathway induced by E2. In general, increased PKCα activity is associated with increased motility and proliferation of cancer cells [39], [40], [41]. In addition, PKCα has been shown to inhibit or facilitate apoptosis of cancer cells [42], [43]. We found that E2 was unable to induce phosphorylation of PKCα in Ishikawa cells, suggesting that E2-induced endometrial cancer growth through signaling pathways other than PKCα.

cAMP is a second messenger that plays a role in intracellular signal transduction of various stimuli. A major function of cAMP in eukaryotes is activation of cAMP-dependent protein kinase (PKA). In the present study, we found that E2 can rapidly activate PKA within 3 min. However, PKA could not be activated by E2-BSA that is unable to traverse the plasma membrane, suggesting that E2 activate PKA not through the membrane-based ER-a36 in Ishikawa cells.

The MAPK family consists of ERK, JNK and P38. ERK plays an essential role in cell growth. Increasing evidence has shown that non-genomic activation of the ERK1/2 by estrogen in both breast and endometrial cancer cells [44], [45], [46]. Previous studies have shown that the ERK1/2 can be efficiently activated by protein kinase C [47], [48]. Here, we found that ER-α36 mediates E2-induced activation of the MAPK/ERK pathway via PKCδ in Ishikawa cells.

Cyclin D1 is a key regulator of cell cycle progression through the G1 phase that, upon its induction, binds and regulates cyclin-dependent kinases (cdks) 4 or 6. The cyclin D1/cdk complexes are capable of phosphorylation substrates essential to promote cell cycle [21], [49], [50]. Cyclin D1 plays an important role in the pathogenesis of endometrial hyperplasia [18], [51], [52]. Previous studies have demonstrated that PKCδ and MAPK/ERK pathways up-regulate cyclin D1 protein expression [19], [51], [53]. Here, we demonstrated that E2 induced cyclin D1 expression through ER-α36-mediated activation of the PKCδ/ERK signaling pathway.

In summary, our results indicate that extra-nuclear ER-α36 mediates the non-genomic estrogen signaling pathways in Ishikawa cells and suggest ER-α36 is a novel and important player in endometrial carcinogenesis.

## Materials and Methods

### Materials and Reagents

All chemicals and reagents were purchased from Sigma unless otherwise indicated. Anti-phospho-PKCα (ser^657^) antibody was purchased from Upstate (Temecula, CA). Antibodies for PKCα, phospho-PKCδ (Thr^505^), PKCδ, phospho-ERK1/2 (Thr^202^/Tyr^204^) and ERK1/2 were purchased from Cell Signaling (Beverly, MA). Antibodies against ER-α66, cyclin D1, cdk4 and β-actin were purchased from Santa Cruz Biotechnology (Santa Cruz, CA). The ER-α36 specific antibody against the 20 unique amino acid at the C-terminal of ER-α36, ER-α36 interference plasmid and control plasmids were described before [8], [15]. Hoechst 33258, MTT, U0126, HBDDE, Rottlerin, Bisindolylmaleimide and Forskolin were purchased from Calbiochem (La Jolla, CA). PepTag Assay kit for Non-Radioactive Detection of cAMP-Dependent Protein Kinase was obtained from Promega (Madison, WI).

### Cell Culture and Cell Lines

The human endometrial cancer cell line Ishikawa was obtained from Dr. Li-Hui Wei (Peking University People's Hospital, Beijing) and cultured in Dulbecco's modified Eagle's medium (Gibco-BRL, USA) with 10% fetal calf serum (Hyclone, UT), 5 ug/ml insulin, and maintained at 37°C in a humidified atmosphere of 5% CO_2_. We established stable Ishikawa cell line transfected with an ER-α36 shRNA expression vector (Ishikawa/RNAiER36), an ER-α66 shRNA expression vector (Ishikawa/RNAiER66) and the empty expression vector (Ishikawa/V). Briefly, the shRNA expression vector pRNAT-U6.1/Neo plasmid containing the shRNA against the 3′UTRs of ER-α36 and ER-α66, respectively and the empty expression vector were transfected into Ishikawa cells with Lipofectamine 2000 (Invitrogen, Carlsbad, CA) according to the manufacturer's instruction. Forty-eight hours after transfection, cells were re-plated and selected with 500 ug/ml of G418 for two weeks. The medium was changed every three days until colonies appeared. Clones were pooled and expanded for further analysis.

### Confocal Microscopy

The cellular localization of ER-α36 and ER-α66 were determined by the indirect immunofluorescence assay. Ishikawa cells cultured on sterile glass coverslips were fixed with 4% paraformaldehyde in PBS for 10 min. After permeabilized with 0.4% Triton X-100 for 10 min at room temperature, cells were blocked in 4% BSA-supplemented PBS for 1 hour and incubated overnight at 4°C with anti-ER-α36-specific antibody or anti-ER-α66 antibody. After three washes in PBS, the cells were labeled with FITC-conjugated secondary antibody. Hoechst 33258 was subsequently added for nuclear staining. Microscopic analysis was performed using a confocal laser-scanning microscope (Zeiss LSM 710 META, Germany).

### Western Blot Analysis

Western blotting was performed as described previously [17]. Cells maintained in phenol-red-free DMEM (Gibcol-BRL, USA) with 2.5% dextran charcoal-stripped fetal calf serum (Biochrom AG, Germany) for 48–72 h were switched to medium without 12 h before treatment with the agents indicated. The cells were collected in ice-cold PBS, and the cell extracts were prepared in RIPA buffer with proteinase inhibitor cocktail from Sigma (St.Louis, MO). The protein concentrations of the cell lysates were determined and boiled with gel-loading buffer for 10 min at 100°C. Samples containing 30 ug of total protein were electrophoresed on 10% SDS-polyacrylamide gels and transferred to PVDF membrane (Millipore, Temecula, CA). The membranes were probed with appropriate primary antibodies and visualized with the corresponding secondary antibodies and the enhanced chemiluminescence detection system (Amersham, Piscataway, NJ).

### RT-PCR

Total RNA was extracted by TRIzol reagent (Invitrogen, Carlsbad, CA). Total RNA (2 ug) was used for production of the first strand cDNA by a reverse transcriptase mixture (Takara, Dalian, P.R.China). The PCR primer sets were listed as the following: ER-α36: forward primer: 5′-CAAGTGGTTTCCTCGTGTCTAAAGC-3′; reverse primer 5′-GTT GAGTGTTGGTTGCCAGG-3′. ER-α66: forward primer: 5′-CACTCAACAGCGT GTCTCCGA-3′; reverse primer: 5′-CCAATCTTTCTCTGCCACCCTG-3′. GAPDH (control): forward primer: 5′-ACGGATTTGGTCGTATTGGG-3′; reverse primer: 5′-TGATTTTGGAGGGATCTCGC-3′. PCR fragments were visualized on a 2% agarose gel stained with ethidium bromide.

### PKA Measurement

Cell lysates were assayed immediately after lyses using the PepTag Assay kit Promega, Madison, WI) to assess the activities of cAMP-dependent protein kinases according to the manufacturer's instructions. Briefly, lysates were prepared from treated cells and incubated with a fluorescently labeled PKA peptide substrate. Phosphorylation of the peptide by activated PKA resulted in a change in its electrophoretic mobility that reflects the relative PKA activity. The agarose gels were subjected to electrophoresis at 110 V for 20 min and visualized under UV light. The upper band is non-phosphorylated substrate peptide, the lower most band is substrate phosphorylated by PKA.

### MTT Assay

Cell proliferation was analyzed using the 3-(4, 5-dimethylthiazol-2-yl)-2, 5- diphenyltrazolium bromide (MTT) assay. Briefly, cells were seeded in a 96-well dish to a final concentration of 1×10^4^ cells/well and incubated in DMEM medium containing 10% FCS for 24 h. Cells were then cultured in phenol red-free medium containing 2.5% charcoal-stripped FCS (Biochrom AG, Berlin, Germany) with the indicated treatments. Medium was removed and fresh medium was added to each well along with 20 ul of MTT solution (5 mg/ml). After 4 h incubation, 150 ul of DMSO were added to each well. The plates were read at wavelength of 490 nm using a microplate reader (Bioteck Powerwave™, USA). Eight duplicate wells were used for each treatment, and experiments were repeated three times.

### Statistical analysis

Statistical analysis was performed with the paired-samples *t*-test, or ANOVA followed by the Student-Newman-Keuls testing to determine differences in means. A level of *P*<0.05 was considered statistically significant. All statistical tests were three-sided.
